# 1-Chloro­methyl-1,4-diazo­niabicyclo­[2.2.2]octane bis­(hexa­fluoro­phosphate)

**DOI:** 10.1107/S1600536811000390

**Published:** 2011-01-12

**Authors:** Run-Qiang Zhu

**Affiliations:** a Ordered Matter Science Research Center, College of Chemistry and Chemical Engineering, Southeast University, Nanjing 211189, People’s Republic of China

## Abstract

In the crystal structure of the title compound, C_7_H_15_ClN_2_^2+^·2PF_6_^−^, the cations and anions are linked by inter­molecular N—H⋯F hydrogen bonds.

## Related literature

For general background to ferroelectric metal-organic frameworks, see: Fu *et al.* (2009 ▶); Ye *et al.* (2006 ▶); Zhang *et al.* (2008 ▶, 2010 ▶).
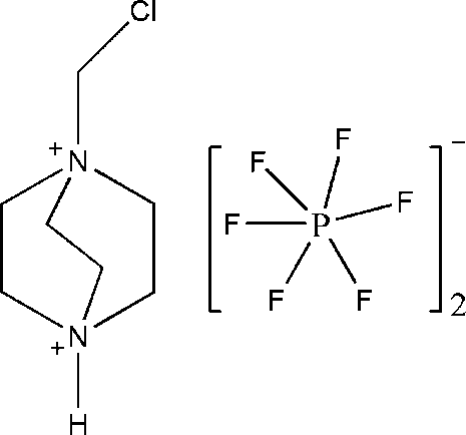


## Experimental

### 

#### Crystal data

C_7_H_15_ClN_2_^2+^·2PF_6_^−^
*M* *_r_* = 452.6
Orthorhombic, 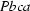

*a* = 14.414 (8) Å
*b* = 12.976 (7) Å
*c* = 16.115 (9) Å
*V* = 3014 (3) Å^3^
*Z* = 8
Mo *K*α radiation
μ = 0.60 mm^−1^
*T* = 293 K
0.30 × 0.25 × 0.20 mm

#### Data collection

Rigaku SCXmini diffractometer
Absorption correction: multi-scan (*CrystalClear*; Rigaku, 2005 ▶) *T*_min_ = 0.836, *T*_max_ = 0.888
30798 measured reflections
3447 independent reflections
3197 reflections with *I* > 2σ(*I*)
*R*_int_ = 0.050

#### Refinement

*R*[*F* ^2^ > 2σ(*F* ^2^)] = 0.056
*wR*(*F* ^2^) = 0.146
*S* = 1.24
3447 reflections
217 parameters
H-atom parameters constrained
Δρ_max_ = 0.58 e Å^−3^
Δρ_min_ = −0.46 e Å^−3^

### 

Data collection: *CrystalClear* (Rigaku, 2005 ▶); cell refinement: *CrystalClear*; data reduction: *CrystalClear*; program(s) used to solve structure: *SHELXS97* (Sheldrick, 2008 ▶); program(s) used to refine structure: *SHELXL97* (Sheldrick, 2008 ▶); molecular graphics: *SHELXTL* (Sheldrick, 2008 ▶); software used to prepare material for publication: *SHELXL97*.

## Supplementary Material

Crystal structure: contains datablocks I, global. DOI: 10.1107/S1600536811000390/fj2377sup1.cif

Structure factors: contains datablocks I. DOI: 10.1107/S1600536811000390/fj2377Isup2.hkl

Additional supplementary materials:  crystallographic information; 3D view; checkCIF report

## Figures and Tables

**Table 1:** Hydrogen-bond geometry (Å, °)

*D*—H⋯*A*	*D*—H	H⋯*A*	*D*⋯*A*	*D*—H⋯*A*
N2—H2*C*⋯F3^i^	0.91	2.26	2.924 (3)	130
N2—H2*C*⋯F4^i^	0.91	2.40	3.073 (3)	131
N2—H2*C*⋯F9^i^	0.91	2.43	3.055 (3)	126

Symmetry code: (i) 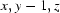.

**Fig. 1.**
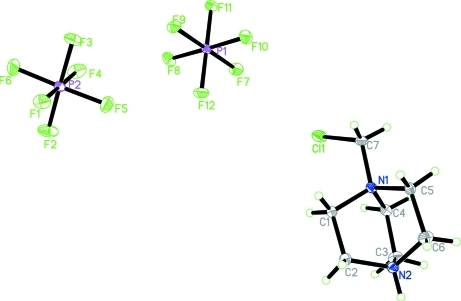
A partial packing diagram of the title compound, with displacement ellipsoids drawn at the 30% probability level.

**Fig. 2.**
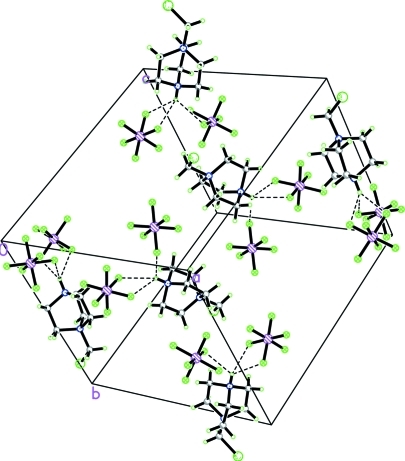
Packing diagram of the title compound, hydrogen bonds are shown as dashed lines.

## supplementary crystallographic information

### Comment

We synthesized the title compound to find ferroelectric material by dielectric measurements of compound as a function of temperature(Fu *et al.* 2009;Ye *et al.*2006; Zhang *et al.*2008; Zhang *et al.*2010). In the range from 190 K to near its melting point(m.p. >452 K), no dielectric anomaly was observed.

Single crystal of the title compound suitable for X-ray diffraction analysis were obtained by evaporating an water solution in 123.5 K. As Fig.1, the compound consists of one 1-(chloromethyl)-1,4- diazabicyclo[2.2.2]octane-1,4-diium cations and two hexafluorophosphate anions. The hydrogen bonds linked one 1-(chloromethyl)-1,4- diazabicyclo[2.2.2]octane-1,4-diium cations and two hexafluorophosphate anions of the another cell as showed in the Fig.2.

### Experimental

1,4-Diazabicyclo[2.2.2]octane(5.6 g,0.05 mol)was dissolved in 20 ml of dichloromethane and the mixture solution was refluxed for 8 h. A white precipitate of 1-(chloromethyl)-1,4-diazabicyclo[2.2.2]octan-1-ium chloride were obtained. The title compound was synthesized by the mixed solution of 1-(chloromethyl)-1,4-diazabicyclo[2.2.2]octan-1-ium chloride(1.97 g, 10 mmol) and hexafluorophosphoric acid(20 mmol). After a few days, colorless block crystals of the title compound were obtained on slow evaporation of the solvent.

### Refinement

Positional parameter of all the H atoms except for H2 were calculated geometrically and the H atoms were set to ride on the C atoms to which they are bonded, with *U*_iso_(H) =1.2Ueq(C). The position of the H atom on N2 was determined from a difference Fourier map and was not refined.

### Crystal data

**Table:**

C_7_H_15_ClN_2_^2+^·2PF_6_^−^	*F*(000) = 1808
*M**_r_* = 452.6	*D*_x_ = 1.995 Mg m^−^^3^
Orthorhombic, *P**b**c**a*	Mo *K*α radiation, λ = 0.71073 Å
Hall symbol: -P 2ac 2ab	Cell parameters from 6697 reflections
*a* = 14.414 (8) Å	θ = 2.5–27.5°
*b* = 12.976 (7) Å	µ = 0.60 mm^−^^1^
*c* = 16.115 (9) Å	*T* = 293 K
*V* = 3014 (3) Å^3^	Prism, colorless
*Z* = 8	0.30 × 0.25 × 0.20 mm

### Data collection

**Table:**

Rigaku SCXmini diffractometer	3447 independent reflections
Radiation source: fine-focus sealed tube	3197 reflections with *I* > 2σ(*I*)
graphite	*R*_int_ = 0.050
CCD Profile fitting scans	θ_max_ = 27.5°, θ_min_ = 2.5°
Absorption correction: multi-scan (*CrystalClear*; Rigaku, 2005)	*h* = −18→18
*T*_min_ = 0.836, *T*_max_ = 0.888	*k* = −16→16
30798 measured reflections	*l* = −20→20

### Refinement

**Table:**

Refinement on *F*^2^	Secondary atom site location: difference Fourier map
Least-squares matrix: full	Hydrogen site location: inferred from neighbouring sites
*R*[*F*^2^ > 2σ(*F*^2^)] = 0.056	H-atom parameters constrained
*wR*(*F*^2^) = 0.146	*w* = 1/[σ^2^(*F*_o_^2^) + (0.0625*P*)^2^ + 3.8206*P*] where *P* = (*F*_o_^2^ + 2*F*_c_^2^)/3
*S* = 1.24	(Δ/σ)_max_ < 0.001
3447 reflections	Δρ_max_ = 0.58 e Å^−^^3^
217 parameters	Δρ_min_ = −0.46 e Å^−^^3^
0 restraints	Extinction correction: *SHELXL97* (Sheldrick, 2008)
Primary atom site location: structure-invariant direct methods	Extinction coefficient: 0.0014 (1)

### Special details

**Table:**

Geometry. All e.s.d.'s (except the e.s.d. in the dihedral angle between two l.s. planes) are estimated using the full covariance matrix. The cell e.s.d.'s are taken into account individually in the estimation of e.s.d.'s in distances, angles and torsion angles; correlations between e.s.d.'s in cell parameters are only used when they are defined by crystal symmetry. An approximate (isotropic) treatment of cell e.s.d.'s is used for estimating e.s.d.'s involving l.s. planes.
Refinement. Refinement of *F*^2^ against ALL reflections. The weighted *R*-factor *wR* and goodness of fit *S* are based on *F*^2^, conventional *R*-factors *R* are based on *F*, with *F* set to zero for negative *F*^2^. The threshold expression of *F*^2^ > σ(*F*^2^) is used only for calculating *R*-factors(gt) *etc*. and is not relevant to the choice of reflections for refinement. *R*-factors based on *F*^2^ are statistically about twice as large as those based on *F*, and *R*- factors based on ALL data will be even larger.

### Fractional atomic coordinates and isotropic or equivalent isotropic displacement parameters (Å^2^)

**Table:**

	*x*	*y*	*z*	*U*_iso_*/*U*_eq_	
P1	0.10243 (5)	0.80778 (6)	0.38500 (5)	0.01982 (19)	
P2	0.25717 (5)	0.96221 (6)	0.14312 (5)	0.01997 (19)	
F8	0.19471 (13)	0.87588 (16)	0.38218 (13)	0.0359 (5)	
F9	0.06202 (13)	0.87478 (14)	0.30870 (11)	0.0293 (4)	
F11	0.05718 (12)	0.88833 (14)	0.44911 (11)	0.0268 (4)	
F10	0.00885 (13)	0.74127 (15)	0.38738 (13)	0.0331 (4)	
F7	0.14303 (13)	0.74224 (16)	0.46061 (11)	0.0343 (5)	
F12	0.14650 (13)	0.72793 (15)	0.32030 (12)	0.0322 (4)	
F3	0.15177 (12)	1.00058 (16)	0.15861 (13)	0.0347 (5)	
F1	0.23000 (15)	0.92628 (16)	0.05253 (11)	0.0363 (5)	
F4	0.28151 (16)	0.99851 (17)	0.23639 (13)	0.0430 (6)	
F2	0.36096 (14)	0.92349 (19)	0.13078 (16)	0.0479 (6)	
F5	0.22953 (16)	0.85130 (15)	0.17743 (14)	0.0421 (5)	
F6	0.28287 (17)	1.07397 (16)	0.11115 (15)	0.0465 (6)	
Cl1	0.09951 (6)	0.45726 (7)	0.44115 (7)	0.0419 (3)	
N1	0.05494 (16)	0.26975 (18)	0.37949 (15)	0.0189 (5)	
N2	0.11243 (16)	0.10322 (19)	0.31629 (15)	0.0206 (5)	
H2C	0.1332	0.0434	0.2933	0.025*	
C5	−0.02655 (19)	0.2006 (2)	0.35656 (18)	0.0207 (6)	
H5A	−0.0654	0.1896	0.4049	0.025*	
H5B	−0.0638	0.2336	0.3141	0.025*	
C1	0.1107 (2)	0.2924 (2)	0.30230 (19)	0.0224 (6)	
H1A	0.1657	0.3316	0.3168	0.027*	
H1B	0.0740	0.3333	0.2641	0.027*	
C3	0.1568 (2)	0.1167 (2)	0.39967 (18)	0.0278 (7)	
H3A	0.2234	0.1239	0.3935	0.033*	
H3B	0.1446	0.0569	0.4341	0.033*	
C7	0.0138 (2)	0.3663 (2)	0.4162 (2)	0.0266 (6)	
H7A	−0.0207	0.3486	0.4660	0.032*	
H7B	−0.0293	0.3964	0.3769	0.032*	
C2	0.1390 (2)	0.1914 (2)	0.2609 (2)	0.0280 (7)	
H2A	0.1080	0.1847	0.2077	0.034*	
H2B	0.2054	0.1908	0.2513	0.034*	
C4	0.1162 (2)	0.2138 (2)	0.44068 (18)	0.0232 (6)	
H4A	0.0804	0.1946	0.4892	0.028*	
H4B	0.1662	0.2588	0.4584	0.028*	
C6	0.0090 (2)	0.0978 (3)	0.3247 (3)	0.0364 (8)	
H6A	−0.0078	0.0434	0.3631	0.044*	
H6B	−0.0188	0.0825	0.2713	0.044*	

### Atomic displacement parameters (Å^2^)

**Table:**

	*U*^11^	*U*^22^	*U*^33^	*U*^12^	*U*^13^	*U*^23^
P1	0.0173 (4)	0.0238 (4)	0.0184 (4)	0.0025 (3)	0.0000 (3)	0.0006 (3)
P2	0.0181 (4)	0.0220 (4)	0.0198 (4)	−0.0001 (3)	0.0000 (3)	−0.0018 (3)
F8	0.0217 (9)	0.0451 (12)	0.0410 (11)	−0.0067 (8)	0.0054 (8)	−0.0014 (9)
F9	0.0340 (10)	0.0322 (10)	0.0218 (9)	0.0108 (8)	−0.0020 (7)	0.0035 (7)
F11	0.0282 (9)	0.0286 (9)	0.0235 (9)	0.0024 (7)	0.0050 (7)	−0.0028 (7)
F10	0.0264 (9)	0.0311 (10)	0.0419 (11)	−0.0074 (8)	0.0020 (8)	−0.0041 (9)
F7	0.0372 (10)	0.0422 (11)	0.0235 (9)	0.0133 (9)	−0.0027 (8)	0.0073 (8)
F12	0.0367 (10)	0.0335 (10)	0.0265 (10)	0.0142 (8)	0.0038 (8)	−0.0031 (8)
F3	0.0199 (9)	0.0403 (11)	0.0440 (11)	0.0016 (8)	0.0030 (8)	−0.0162 (9)
F1	0.0466 (12)	0.0429 (11)	0.0195 (9)	0.0112 (10)	−0.0053 (8)	−0.0082 (8)
F4	0.0560 (14)	0.0420 (12)	0.0310 (11)	0.0127 (10)	−0.0191 (10)	−0.0121 (9)
F2	0.0208 (10)	0.0539 (14)	0.0690 (16)	0.0064 (9)	−0.0004 (10)	−0.0187 (12)
F5	0.0614 (14)	0.0256 (10)	0.0392 (11)	−0.0020 (10)	0.0077 (10)	0.0035 (9)
F6	0.0572 (14)	0.0280 (10)	0.0544 (14)	−0.0099 (10)	0.0148 (11)	0.0055 (10)
Cl1	0.0285 (4)	0.0331 (4)	0.0641 (6)	−0.0071 (3)	0.0121 (4)	−0.0250 (4)
N1	0.0164 (11)	0.0205 (11)	0.0199 (11)	−0.0001 (9)	0.0005 (9)	−0.0011 (9)
N2	0.0191 (12)	0.0200 (11)	0.0227 (12)	0.0001 (9)	0.0003 (9)	−0.0010 (10)
C5	0.0159 (13)	0.0237 (14)	0.0225 (14)	−0.0027 (10)	−0.0020 (10)	−0.0003 (11)
C1	0.0218 (14)	0.0202 (13)	0.0252 (14)	−0.0011 (11)	0.0051 (11)	0.0007 (11)
C3	0.0348 (17)	0.0266 (15)	0.0220 (14)	0.0078 (13)	−0.0052 (12)	0.0007 (12)
C7	0.0204 (14)	0.0213 (14)	0.0382 (17)	0.0025 (11)	0.0046 (12)	−0.0076 (13)
C2	0.0377 (17)	0.0239 (15)	0.0223 (15)	0.0035 (13)	0.0074 (13)	0.0043 (12)
C4	0.0200 (13)	0.0294 (15)	0.0201 (13)	0.0005 (12)	−0.0014 (11)	−0.0014 (11)
C6	0.0190 (15)	0.0282 (16)	0.062 (2)	−0.0040 (13)	−0.0030 (15)	−0.0145 (16)

### Geometric parameters (Å, °)

**Table:**

P1—F7	1.597 (2)	N2—C2	1.501 (4)
P1—F8	1.598 (2)	N2—H2C	0.9100
P1—F12	1.601 (2)	C5—C6	1.518 (4)
P1—F10	1.602 (2)	C5—H5A	0.9700
P1—F11	1.6079 (19)	C5—H5B	0.9700
P1—F9	1.6147 (19)	C1—C2	1.527 (4)
P2—F1	1.582 (2)	C1—H1A	0.9700
P2—F6	1.583 (2)	C1—H1B	0.9700
P2—F2	1.591 (2)	C3—C4	1.538 (4)
P2—F5	1.592 (2)	C3—H3A	0.9700
P2—F4	1.614 (2)	C3—H3B	0.9700
P2—F3	1.618 (2)	C7—H7A	0.9700
Cl1—C7	1.756 (3)	C7—H7B	0.9700
N1—C7	1.507 (4)	C2—H2A	0.9700
N1—C4	1.510 (4)	C2—H2B	0.9700
N1—C1	1.510 (4)	C4—H4A	0.9700
N1—C5	1.524 (3)	C4—H4B	0.9700
N2—C6	1.498 (4)	C6—H6A	0.9700
N2—C3	1.498 (4)	C6—H6B	0.9700
			
F7—P1—F8	90.65 (12)	C6—C5—N1	109.8 (2)
F7—P1—F12	90.40 (11)	C6—C5—H5A	109.7
F8—P1—F12	90.53 (12)	N1—C5—H5A	109.7
F7—P1—F10	90.19 (12)	C6—C5—H5B	109.7
F8—P1—F10	178.98 (12)	N1—C5—H5B	109.7
F12—P1—F10	90.06 (11)	H5A—C5—H5B	108.2
F7—P1—F11	90.26 (11)	N1—C1—C2	109.6 (2)
F8—P1—F11	89.79 (11)	N1—C1—H1A	109.8
F12—P1—F11	179.26 (11)	C2—C1—H1A	109.8
F10—P1—F11	89.62 (11)	N1—C1—H1B	109.8
F7—P1—F9	179.52 (13)	C2—C1—H1B	109.8
F8—P1—F9	88.90 (11)	H1A—C1—H1B	108.2
F12—P1—F9	89.75 (11)	N2—C3—C4	108.6 (2)
F10—P1—F9	90.26 (11)	N2—C3—H3A	110.0
F11—P1—F9	89.59 (10)	C4—C3—H3A	110.0
F1—P2—F6	91.58 (13)	N2—C3—H3B	110.0
F1—P2—F2	91.39 (12)	C4—C3—H3B	110.0
F6—P2—F2	91.64 (14)	H3A—C3—H3B	108.3
F1—P2—F5	89.54 (12)	N1—C7—Cl1	111.8 (2)
F6—P2—F5	178.30 (13)	N1—C7—H7A	109.3
F2—P2—F5	89.61 (13)	Cl1—C7—H7A	109.3
F1—P2—F4	178.20 (13)	N1—C7—H7B	109.3
F6—P2—F4	89.13 (13)	Cl1—C7—H7B	109.3
F2—P2—F4	90.24 (13)	H7A—C7—H7B	107.9
F5—P2—F4	89.71 (13)	N2—C2—C1	109.1 (2)
F1—P2—F3	90.03 (11)	N2—C2—H2A	109.9
F6—P2—F3	89.32 (13)	C1—C2—H2A	109.9
F2—P2—F3	178.26 (14)	N2—C2—H2B	109.9
F5—P2—F3	89.40 (12)	C1—C2—H2B	109.9
F4—P2—F3	88.32 (11)	H2A—C2—H2B	108.3
C7—N1—C4	111.9 (2)	N1—C4—C3	109.6 (2)
C7—N1—C1	111.8 (2)	N1—C4—H4A	109.8
C4—N1—C1	108.7 (2)	C3—C4—H4A	109.8
C7—N1—C5	106.3 (2)	N1—C4—H4B	109.8
C4—N1—C5	109.0 (2)	C3—C4—H4B	109.8
C1—N1—C5	109.0 (2)	H4A—C4—H4B	108.2
C6—N2—C3	110.4 (3)	N2—C6—C5	109.0 (2)
C6—N2—C2	110.1 (3)	N2—C6—H6A	109.9
C3—N2—C2	109.6 (2)	C5—C6—H6A	109.9
C6—N2—H2C	108.9	N2—C6—H6B	109.9
C3—N2—H2C	108.9	C5—C6—H6B	109.9
C2—N2—H2C	108.9	H6A—C6—H6B	108.3

### Hydrogen-bond geometry (Å, °)

**Table:**

*D*—H···*A*	*D*—H	H···*A*	*D*···*A*	*D*—H···*A*
N2—H2C···F3^i^	0.91	2.26	2.924 (3)	130
N2—H2C···F4^i^	0.91	2.40	3.073 (3)	131
N2—H2C···F9^i^	0.91	2.43	3.055 (3)	126

Symmetry codes: (i) *x*, *y*−1, *z*.
